# Outcomes of COVID-19 in Adult Males With Hemophilia A: A Propensity Score-Matched Analysis

**DOI:** 10.7759/cureus.30662

**Published:** 2022-10-25

**Authors:** Meric Mericliler, Gayatri Narayan

**Affiliations:** 1 Hematology and Medical Oncology, Virginia Commonwealth University, Richmond, USA; 2 Hematology and Medical Oncology, Massey Cancer Center, Richmond, USA; 3 Internal Medicine, Saint Vincent Hospital, Worcester, USA

**Keywords:** arteriovenous thromboembolism in sars-cov-2-infected patients, covid coagulopathy, bleeding risk, 30 day mortality, factor viii deficiency, hemophilia-a, covid 19

## Abstract

Background

Hypercoagulability is a major pathologic event in COVID-19. Factor VIII plays an important role in hemostasis, and high levels of factor VIII have been shown to be associated with an increased risk of thrombosis and severe disease. Little is known about the impact of COVID-19 on clinical outcomes in patients with hemophilia A.

Methodology

Retrospective data of adult male patients with COVID-19 with and without hemophilia A were retrieved from the TriNetX database (Cambridge, USA). The 1:1 propensity score-matching was performed to balance baseline characteristics. Patients were matched for age, race, body mass index, and medical comorbidities. Thirty-day outcomes were assessed.

Results

We identified 1,758 patients with pre-existing hemophilia A diagnosis prior to COVID-19 diagnosis and 5,191,908 comparators. After 1:1 propensity score matching, groups were balanced on demographics and comorbidities. All-cause mortality rates were similar between the two groups (HR 0.805; 95% CI 0.467-1.389). The frequency of severe infection, ICU admission, and composite thrombotic events did not differ between the groups. Patients with hemophilia A were hospitalized more frequently than those without a history of hemophilia A (19.2% vs. 14.4%; p<0.05). Additionally, gastrointestinal (GI) bleeding and composite bleeding events occurred more frequently in patients with hemophilia A (3.2% vs. 2.2%; p<0.05 and 4.0% vs. 2.8%; p<0.05, respectively).

Conclusions

The mortality of individuals with hemophilia A due to COVID-19 is comparable to the general population but with higher risks of hospitalization and bleeding.

## Introduction

Background

Hypercoagulability is one of the hallmark pathologic features of COVID-19, and COVID-19-associated coagulopathy (CAC) is an important cause of morbidity and mortality [1]. The exact mechanism of CAC remains elusive. Studies have shown that endothelial cell dysfunction, increased platelet activation, activation of the coagulation, and alterations in the fibrinolytic system may play an important role in the pathogenesis [2]. Elevated factor VIII activity is commonly observed in critically ill patients with COVID-19 [3,4]. Additionally, patients with COVID-19 have been shown to have higher factor VIII activity compared to control patients with non-SARS-CoV-2 pneumonia, suggesting inflammation may not be the sole factor contributing to the elevated factor VIII activity [5]. Elevated factor VIII activity is a predictor of severity and mortality in COVID-19 patients [6].

Little is known about the impact of COVID-19 on clinical outcomes in patients with bleeding disorders. Early observational studies argued that patients with congenital bleeding disorders (CBD) tend to have similar disease severity or even less severe disease compared to the rest of the population [7-9]. However, these studies were not comparative and included only a limited number of patients.

Objective

The aim of this study was to investigate the clinical outcomes of COVID-19 in patients with hemophilia A using a propensity score-matched analysis in a multi-institutional research network database.

## Materials and methods

Study design and setting

This was a multicenter retrospective cohort study, which assessed the outcomes of COVID-19 in patients with hemophilia A. Retrospective data of patients with COVID-19 with and without hemophilia A was retrieved from the TriNetX database (Cambridge, USA), a global health collaborative clinical research platform collecting anonymous data from the electronic medical records of 90 healthcare organizations. The TriNetX database has been previously described in the literature [10]. 

Participants

We identified two groups: (1) Males aged 18 years and older with hemophilia A who tested positive for SARS-CoV-2 and (2) Males aged 18  years and older without hemophilia A who tested positive for SARS-CoV-2. We included patients who tested positive for SARS-CoV-2 between December 1, 2020, and September 20, 2022. Cases were identified using relevant diagnostic codes. We excluded females, patients younger than 18 or older than 90 years of age, and those diagnosed with acquired coagulation factor deficiencies.

The study was exempt from institutional review board approval because we utilized deidentified patient records on the TriNetX platform.

Variables and outcome measures

The 1:1 propensity score matching was performed to balance baseline characteristics. Variables used in the propensity matching represent the established risk factors for severe COVID-19. Patients were matched for age, race, overweight/obesity, ischemic heart disease, cerebrovascular disease, peripheral vascular disease, heart failure, atrial fibrillation, venous thromboembolism, tobacco use disorder, alcohol-related disorders, liver disease, HIV infection, chronic lower respiratory disease, chronic kidney disease, hypertension, diabetes mellitus, and neoplasm. 

The primary outcomes of our study included all-cause mortality, severe COVID-19 (composite outcome of ICU admission, mechanical ventilation, or death), and the composite of thrombotic events within 30 days after infection. The secondary outcomes included hospitalization, ICU admission, need for mechanical ventilation, need for renal replacement therapy, venous thromboembolism, ischemic stroke, myocardial infarction, intracranial hemorrhage (ICH), gastrointestinal bleeding, and the composite of intracerebral hemorrhage and gastrointestinal bleeding. All examined outcomes were assessed within 30 days after the COVID-19 diagnosis. 

Outcomes were defined by using the appropriate Logical Observation Identifiers Names and Codes (LOINCs), International Classification of Diseases, Tenth Revision, Clinical Modification (ICD‐10‐CM), Current Procedural Terminology (CPT), and National Drug File Reference Terminology (NDF-RT) codes. The details of the diagnosis and laboratory test codes for the outcome measures are described in Table 1. 

**Table 1:** List of the ICD-10, LOINC, CPT, and NDF-RT codes used for obtaining data from the database. ICD-10: 10th revision of the International Statistical Classification of Diseases and Related Health Problems; CPT: Current Procedural Terminology; LOINC: Logical Observation Identifiers Names and Codes; NDF-RT: National Drug File Reference Terminology; TIA: transient ischemic attack.

ICD-10	
COVID-19	U07.1, U07.2
Hereditary factor VIII deficiency	D66
Acquired coagulation factor deficiency	D68.4
Overweight and obesity	E66
Essential hypertension	I10
Alcohol related disorders	F10
Nicotine dependence	F17
Chronic kidney disease	N18
Ischemic heart diseases	I20-I25
Heart failure	I50
Disorders of lipoprotein metabolism	E78
Atrial fibrillation and flutter	I48
Peripheral vascular disease	I73
Diseases of liver	K70-K77
Chronic lower respiratory diseases	J40-J47
Diabetes mellitus	E08-E11
Arterial embolism and thrombosis	I74
Transient cerebral ischemic attack and related syndromes	G45
Cerebrovascular disease	I60-69
Neoplasm	C00-D49
Chronic kidney disease	N18
HIV infection	B20
Pulmonary embolism	I26
Other venous thromboembolism	I82
Renal Replacement Therapy	Z99.2
Mechanical ventilation	5A19
Gastrointestinal bleeding	K92.0, 92.1, 92.2
Intracranial hemorrhage	I60-62
Ischemic stroke and TIA	G45, I63, I67
Composite outcome for thrombotic events	G45, I63, I67, I74, I26, I81, I82, I21, I22
Venous thromboembolism	I26, I81, I82
Myocardial infarction	I21, I22
LOINC	
Positive testing for SARS-CoV2	9088, 94500-6, 94309-2, 94534-5, 94565-9, 94759-8, 94558-4, 94316-7, 94559-2, 96119-3, 95608-6, 94763-0, 94533-7, 94760-6, 94845-5, 96763-8, 95406-5, 94314-2, 96123-5, 97097-0, 94757-2, 95409-9, 94308-4, 94307-6
Factor VIII activity in plasma	3209-4
Positive testing for HIV	56888-1, 7918-6
White (race)	2106-3
Black or African American	2054-5
CPT	
Hospitalization	1013660, 1013659, 1013699 99221-99226; 99231-99233
Critical care services	99291, 99292, 1013729, 1014309
Renal replacement therapy	90945, 90947, 90935, 90937, 90940, 36800
Mechanical ventilation	94002, 94003, 31500, 1015098, 5A19
NDF-RT	
Antiplatelet therapy	BL117
Anticoagulant therapy	BL110

Statistical methods

Descriptive statistics were presented as mean and standard deviation or percentages. The TriNetX platform was used to perform 1:1 propensity score matching with the use of logistic regression. Outcome analysis was performed before and after propensity score matching. Kaplan-Meier curves and log-rank tests were used to investigate the differences in all-cause mortality between groups. Risk Ratios (RR) with 95% confidence intervals (CI) for 30-day outcomes were calculated for each outcome. A p-value of <0.05 was considered statistically significant. All statistical analyses were conducted on the TriNetX platform. 

## Results

Baseline demographic and clinical characteristics

We identified 1,758 patients with pre-existing hemophilia A diagnosis prior to COVID-19 diagnosis (mean age 51.3 years; 73.1% white) and 5,191,908 comparators (mean age 49.7 years; 55.2% white). Among the patients without hemophilia A, 5,191,102 patients were included in the statistical analysis. Patients with hemophilia A had higher rates of comorbidities (Table 2). In relation to this, patients with anticoagulation and antiplatelet use were higher in the hemophilia group (20% vs. 7% and 15% vs. 7%, respectively).

**Table 2:** Baseline characteristics of the patients Values are the number (%) of patients. S. Diff = standardized difference; BMI = body mass index; VTE = venous thromboembolism; PE = Pulmonary embolism; CLRD: chronic lower respiratory tract disease.

	Before matching				After matching			
	hemophilia cohort (n=1,758)	non-hemophilia cohort (n=5,191,908)	p-value	S. Diff	hemophilia cohort (n=1,758)	non-hemophilia cohort (n=1,758)	p-value	S. Diff
Demographics								
Age (years)	51.3 ± 19.8	49.7 ± 19	0.0004	0.0824	51.3 ± 19.8	52.6 ± 19.3	0.0429	0.0683
White (%)	73.1%	55.2%	< 0.0001	0.3811	73.1%	75.6%	0.0967	0.056
Black or African American (%)	12.5%	13.1%	0.4518	0.0181	12.5%	12.2%	0.7975	0.0087
Comorbidities								
Hypertension (%)	52.2%	24.0%	< 0.0001	0.607	52.2%	56.7%	0.0075	0.0903
Diabetes mellitus (%)	24.6%	11.1%	< 0.0001	0.3577	24.6%	25.8%	0.3928	0.0288
Nicotine dependence (%)	26.1%	9.5%	< 0.0001	0.4458	26.1%	28.3%	0.1498	0.0486
Overweight and obesity (%)	23.6%	10.7%	< 0.0001	0.346	23.6%	27.3%	0.0119	0.085
Ischemic heart disease (%)	27.0%	9.9%	< 0.0001	0.4517	27.0%	29.7%	0.0786	0.0593
Peripheral vascular disease (%)	9.8%	2.8%	< 0.0001	0.2885	9.8%	9.6%	0.8643	0.0058
Heart failure	17.9%	4.7%	< 0.0001	0.4288	17.9%	18.7%	0.5416	0.0206
Atrial fibrillation	11.0%	5.2%	< 0.0001	0.2169	11.0%	12.3%	0.2477	0.039
Cerebrovascular disease (%)	24.1%	5.3%	< 0.0001	0.5507	24.1%	24.4%	0.8133	0.008
CLRD (%)	32.4%	10.8%	< 0.0001	0.545	32.4%	33.6%	0.4732	0.0242
VTE, excluding PE (%)	12.5%	2.2%	< 0.0001	0.4041	12.5%	12.8%	0.7606	0.0103
PE %	6.1%	1.0%	< 0.0001	0.2781	6.1%	4.7%	0.0616	0.0631
Chronic kidney disease (%)	15.9%	5.8%	< 0.0001	0.3303	15.9%	17.0%	0.4128	0.0276
Liver disease (%)	31.6%	4.9%	< 0.0001	0.735	31.6%	32.3%	0.6642	0.0146
Alcohol related disorders (%)	11.4%	4.1%	< 0.0001	0.2771	11.4%	11.1%	0.831	0.0072
HIV infection (%)	13.1%	0.7%	< 0.0001	0.5037	13.1%	11.7%	0.1834	0.0449

Outcome analysis

Before matching, there was no difference in all-cause mortality between the two groups (HR 1.027; 95% CI 0.688-1.533; p=0.682). Both groups had similar rates of composite outcome, severe COVID-19, and mechanical ventilation. Patients with hemophilia A were more likely to be hospitalized and require ICU level of care within 30 days after infection. Composite thrombotic events, venous thromboembolism and ischemic cerebrovascular events were significantly more frequent in patients with hemophilia A. Additionally, higher rates of gastrointestinal bleeding and **ICH** were observed in patients with hemophilia A. 

**Figure 1:**
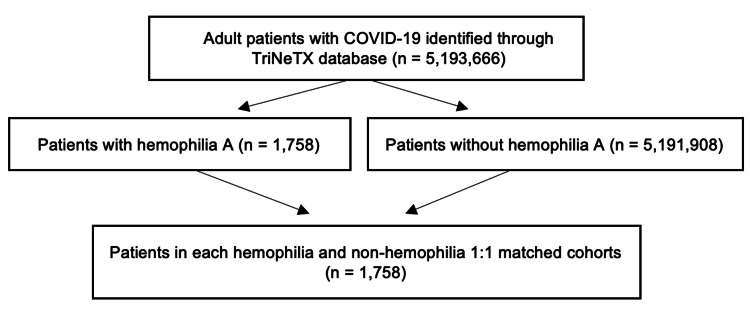
Patient flow diagram

After propensity score matching, there were 1,758 patients included in each cohort (Figure 1). Both groups were well-balanced on demographics and comorbidities. All-cause mortality was comparable between the two groups (HR 0.805; 95% CI 0.467-1.389). Patients in the hemophilia group and comparators had similar rates of severe COVID-19 infection (RR 0.85; 95% CI 0.612-1.184). Patients with hemophilia A were hospitalized more frequently than those without a history of hemophilia A (RR 1.34; 95% CI 1.151-1.55), but the rates of ICU admission were comparable between groups. No differences between groups were seen in venous thromboembolism, composite thrombotic events, myocardial infarction, and ischemic cerebrovascular events. GI bleeding and composite bleeding events occurred more frequently in patients with hemophilia A (RR 1.50; 95% CI 1.000-2.249 and RR 1.45 95% CI 1.013-2.073, respectively). The results are presented in Table 3. 

**Table 3:** Outcomes at 30 days following COVID-19 diagnosis among patients with hemophilia A compared to patients without hemophilia A Except where indicated otherwise, values are the number (%) of patients. RR = relative risk; 95% CI = 95% confidence interval. *statistically significant outcome; AKI: acute kidney injury; RRT: renal replacement therapy.

	Before matching			After matching		
	hemophilia cohort (n=1,758)	non-hemophilia cohort (n=5,191,102)	RR (95% CI)	hemophilia cohort (n=1,758)	non-hemophilia cohort (n=1,758)	RR (95% CI)
All-cause mortality	24 (1.4%)	53,802 (1.0%)	1.31 (0.885, 1.960)	24 (1.4%)	28 (1.6%)	0.86 (0.499, 1.473)
Severe infection	63 (3.6%)	140,711 (2.7%)	1.32 (1.038, 1.685)*	63 (3.6%)	74 (4.2%)	0.85 (0.612, 1.184)
ICU admission	41 (2.3%)	86,847 (1.7%)	1.39 (1.030, 1.887)*	41 (2.3%)	51 (2.9%)	0.80 (0.536, 1.206)
Hospitalization	338 (19.2%)	448,739 (8.6%)	2.23 (2.021, 2.448)*	338 (19.2%)	253 (14.4%)	1.34 (1.151, 1.551)*
Mechanical ventilation	22 (1.3%)	55,575 (1.1%)	1.17 (0.772, 1.771)	22 (1.3%)	25 (1.4%)	0.88 0.498, 1.555)
Composite thrombotic events	122 (6.9%)	164,406 (3.2%)	2.19 (1.847, 2.601)*	122 (6.9%)	127 (7.2%)	0.96 (0.756, 1.221)
Ischemic cerebrovascular events	45 (2.6%)	64,522 (1.2%)	2.06 (1.544, 2.749)*	45 (2.6%)	48 (2.7%)	0.94 (0.628, 1.400)
Venous thromboembolism	74 (4.2%)	60,864 (1.2%)	3.59 (2.873, 4.489)*	74 (4.2%)	72 (4.1%)	1.03 (0.748, 1.412)
Myocardial infarction	24 (1.4%)	49,185 (0.9%)	1.44 (0.968, 2.144)	24 (1.4%)	28 (1.6%)	0.86 (0.499, 1.473)
Intracranial hemorrhage	15 (0.9%)	18,293 (0.04%)	2.42 (1.463, 4.009)*	15 (0.9%)	13 (0.7%)	1.15 (0.551, 2.418)
Gastrointestinal bleeding	57 (3.2%)	40,335 (0.8%)	4.17 (3.232, 5.389)*	57 (3.2%)	38 (2.2%)	1.50 (1.000, 2.249)*
Composite bleeding	71 (4.0%)	57,732 (1.1%)	3.63 (2.892, 4.562)*	71 (4.0%)	49 (2.8%)	1.45 (1.013, 2.073)*
AKI requiring RRT	10 (0.6%)	13,038 (0.3%)	2.27 (1.221, 4.204)*	10 (0.6%)	17 (1%)	0.59 (0.270, 1.281)

## Discussion

The COVID-19 pandemic has affected more than 600 million people worldwide to date [11]. One of the hallmarks of COVID-19 infection is coagulopathy, which is associated with increased morbidity and mortality [12]. The pathophysiology of COVID‐19 coagulopathy is complex and not yet fully elucidated. Studies have shown that endothelial cell dysfunction, coagulation activation, and dysregulated fibrinolytic system play a major role in the pathogenesis of CAC [2,13]. Elevated factor VIII levels have been consistently observed in patients with COVID-19 and are a predictor of severity and mortality in COVID-19 patients [4,6]. Routine pharmacologic thromboprophylaxis is associated with improved outcomes in COVID-19, including mortality, and is recommended for hospitalized patients [14]. This poses a unique challenge, particularly for those with bleeding disorders requiring admission to the hospital due to inherent increased bleeding risk.

Studies have shown that the COVID-19 pandemic had a negative impact on lifestyle behaviors, routine care, and replacement therapies in patients with CBD [15-18]. However, there are only a limited number of clinical studies that have investigated the outcomes of COVID-19 in patients with bleeding disorders, and it is not known whether patients with bleeding disorders are more or less prone to the severe form of infection. Álvarez Román et al. investigated the outcomes of COVID-19 in 42 patients with CBD [7]. The majority of the patients had hemophilia A and were on prophylactic treatment. All patients who became infected with SARS-CoV-2 had mild disease, with none requiring specific treatment for infection. Another retrospective study reported outcomes of COVID-19 in nine patients with CBDs, of whom most had severe hemophilia. Among the nine patients, only one patient had severe disease, two experienced bleeding, and one experienced thrombosis [9]. Another retrospective, cross-sectional study from Iran, investigated the prevalence and outcome of COVID-19 in patients with CBD. They included nine patients with CBDs who had COVID-19. Only three patients required hospital admission, and there was no death [8].

Our results show that patients with hemophilia A were likely to have a severe infection, thrombotic complications, and bleeding events. However, patients with hemophilia A had a higher prevalence of comorbidities that were associated with severe COVID-19. Certain cardiovascular risk factors, such as hypertension as well as HIV and viral hepatitis infections were more prevalent in hemophilia patients in comparison with patients without hemophilia [19-22]. Therefore, to minimize bias, the baseline characteristics between the two groups were balanced using the propensity score-matched analysis. After matching, the rates of all-cause mortality, severe infection, or thrombotic events did not differ between the groups. We have shown that patients with hemophilia A were hospitalized more frequently compared to the general population. The reason for higher rates of hospitalization is unclear, but increased prevalence and risk of bleeding as well as physicians’ preference are potential causes. Although the rates of severe disease were similar in our study, the definition of severe COVID-19 varies across guidelines and institutions [23,24]. Hence, we acknowledge that the difference in the hospitalization rate might still have been due to the higher prevalence of severe COVID-19 in patients with hemophilia A. 

We also showed that patients with hemophilia A had significantly more GI bleeding and composite bleeding events within 30 days after infection. Patients with hemophilia A have an increased risk of developing severe bleeding compared to the general population, and anticoagulation further increases the risk of bleeding. However, there is only limited evidence about the outcomes of prophylactic anticoagulation in hemophilia patients with COVID-19, and the impact of anticoagulation in patients with bleeding disorders needs to be addressed in further studies.

Limitations

The limitations of our study include retrospective design and the utilization of large datasets which potentially includes missing data. However, the TriNetX database has been shown to be reliable and have referential integrity [25]. Additionally, because of the reliance on diagnostic and procedure codes in data extraction, outcomes in different severity of hemophilia A were not evaluated. We excluded females because the majority of female patients are asymptomatic carriers and likely to carry the same ICD-10 codes as patients with hemophilia A, which would lead to bias.

It is important to note that the current study did not evaluate the impact of treatments for COVID-19. Thromboprophylaxis, corticosteroids, and antiviral therapies have been shown to improve disease-related outcomes in patients with COVID-19 [26]. Hence, differences in treatments between the groups may influence the outcomes. Additional research with individual patient data is required to address the impact of COVID-19-directed therapies in patients with bleeding disorders.

## Conclusions

Our findings show that the mortality rate due to COVID-19 in individuals with hemophilia A is comparable to the general population. Additionally, patients with hemophilia A were not at higher risk for severe infection and thromboembolic events. However, patients with hemophilia A had higher rates of hospitalization and bleeding. Further research is warranted to fully characterize risks.
